# Student–teacher gender congruence and student performance: The role of context

**DOI:** 10.1007/s11218-024-09922-2

**Published:** 2024-06-03

**Authors:** Laura Doornkamp, Frank Doornkamp, Lotte D. Van der Pol, Sandra Groeneveld, Judi Mesman, Marleen G. Groeneveld

**Affiliations:** 1 University of Applied Sciences Saxion, Handelskade 75, 7417 DH Deventer, The Netherlands; 2 Biomedical Data Sciences, Leiden University Medical Center, Eindhovenweg 20, 2333ZC Leiden, The Netherlands; 3 Leiden University College, University Leiden, Turfmarkt 99, 2511DC Den Haag, The Netherlands; 4 Institute of Public Administration, University Leiden, Turfmarkt 99, 2511DC Den Haag, The Netherlands

**Keywords:** Student–teacher gender congruence, Student performance, Educational level, Religiousness, Location, Gender stereotypes

## Abstract

Student–teacher gender congruence is suggested to be related to increased student performance, but little is known about the contexts in which these effects occur. Based on literature on gender stereotypes this study hypothesizes different effects of student–teacher gender congruence for male and female students across school subjects and in different educational contexts. Using administrative data of secondary schools in The Netherlands (*N* > 50,000), this study examined to what extent student–teacher gender congruence is associated with male and female students’ performance in the subjects math, physics, Dutch language, and French language. Further this study explored the role of students’ educational level, schools’ religiousness, and schools’ location in these relations. As expected, we found that gender congruence was positively related to female students’ performance in math and physics and to male students’ performance in Dutch language and French language. However, the role of educational context differed for male and female students across subjects and lacked a clear pattern that corresponded to the gender stereotypes hypotheses. This study emphasizes that effects of student–teacher gender congruence can differ in magnitude and direction in different contexts, encouraging future research to use qualitative methods to examine how context influences the role of gender in education.

## Introduction

The education system is intended to provide equal opportunities to all students. Ideally, student performance is a product of talent, ambition, and effort in school, independent of ascriptive background factors such as gender and socioeconomic background (Montt, 2011). Nevertheless, students’ performance is often linked to factors, including students’ and teachers’ gender. For instance, female students’ math performance is found to be related to the presence of female math teachers (e.g., Marx & Roman, 2002), and the deteriorated relative performance of male students is suggested to be linked to the increasing feminization of the education system (Holmlund & Sund, 2008; Neugebauer et al., 2011). Based on representative bureaucracy theory (Song, 2018; Zhang, 2019) and role-model explanations (Carrington et al., 2008; Herrmann et al., 2016), congruence between teachers’ gender and students’ gender is assumed to be positively associated with student performance. However, empirical evidence for the relation is ambiguous. Previous research showed positive effects of student–teacher gender congruence (e.g., Muralidharan & Sheth, 2016), negative effects (e.g., Lindahl, 2016), and null effects (e.g., Holmlund & Sund, 2008).

These ambiguous findings question the assumption that the relation between student–teacher gender congruence and student performance is similar across different subjects and educational contexts (An et al., 2021; Tsai et al., 2017). The relation between student–teacher gender congruence and student performance can be dependent on the extent to which gender is perceived as relevant to the context at hand, i.e., perceived gender salience (e.g., Keiser et al., 2002). In schools, gender may be salient in subjects such as math, physics, and languages because these are the subjects in which male and female students’ performance is gender stereotyped as fundamentally different (men are stereotyped as naturally talented in math and physics, while women are stereotyped as naturally talented in languages) (Guimond & Roussel, 2000). In these subjects with gender stereotypes connected to them, gender can become an important characteristic for students’ identity, interactions, and performance (Hilliard & Liben, 2010; Kiefer & Sekaquaptewa, 2007; Shih et al., 1999). As a result, the performance of female students in math and/or physics may be negatively impacted when they have a female teacher because the male math teacher confirms the stereotype that women are bad at math and physics (Bussey & Bandura, 1999; Doornkamp et al., 2019). Similarly, the performance of male students in languages such as Dutch and French may be negatively impacted when they have a female teacher because a female teacher confirms the stereotype that men are bad at languages (Dee, 2007; Pansu et al., 2016; Van Loo et al., 2013).

Apart from the type of subject, educational contexts may affect the relative strength of gender stereotypes and perceived gender salience (King et al., 2021). Although the relative strength of gender stereotypes in different educational contexts received little research attention to date, previous research indicates that the strength of gender stereotypes varies among social contexts (Miller et al., 2015; Nosek et al., 2009; O’Brien et al., 2015). Similar to social contexts, educational contexts (i.e., schools) have their own sets of norms and values on what is appropriate and inappropriate behavior (Weinstein, 1991). These norms and values can be gender related. Some schools may have traditional views on what is appropriate behavior for men and women (for instance school uniforms in which in female students must wear skirts) whereas other schools may strive for equal norms for male and female students. Educational contexts in which traditional views on appropriate behavior for men and women are present, teachers’ and students’ gender stereotypes may be relatively strong. Previous research indicates that gender stereotypes may be relatively strong in lower educational school levels (Trusty et al., 2000; Turner et al., 2019), in religious schools (Schulze & Tomal, 2005), and in schools located in less-populated areas (Garcia-Retamero et al., 2011). In contexts in which gender stereotypes are relatively strong, gender may be relatively salient and, therefore, student–teacher gender congruence might have a stronger association with student performance.

In this study we examine the relation between teachers’ gender and male and female students’ performance in four secondary school subjects in the Netherlands. We aim to answer the following research questions:

*Research question 1:* To what extent is student-teacher gender congruence associated with male and female students’ performance in math, physics, Dutch language and French language?

*Research question 2*: What is the role of students’ educational level, schools’ religiousness, and schools’ location in the relation between student-teacher gender congruence and students’ performance?

We retrieved student data from secondary schools’ student administration systems in The Netherlands enabling us to examine effects of student–teacher gender congruence for over 50,000 students and their teachers nested in more than 70 schools. Answers to our research questions contribute to previous research on student–teacher gender congruence and student performance that thus far rarely examined its effects beyond the ‘female students in math’ context. To our knowledge only a handful of studies extended the focus to both female and male students’ performance in math (Lindahl, 2016; Zhang, 2019) or to both female and male students’ performance in a science, technology, engineering, and math (STEM) subject and language subject (Dee, 2007; Holmlund & Sund, 2008; Neugebauer et al., 2011). The few studies that did focus on both male and female students in multiple subjects relied on data collected in respectively 1988, 1997–2004, and 2001. Our research adds to this literature by examining performance effects of student–teacher gender congruence for male and female students in math, physics, and Dutch and French language based on current register data (2011–2021). Moreover, this study is the first to explore the role of educational level, religiousness and school location in the relation between student–teacher gender congruence and students’ performance. Thereby, this study contributes to the understanding of where and when effects of student–teacher gender congruence may occur.

## Theoretical framework

### Student–teacher gender congruence and students’ performance

The relation between student–teacher gender congruence and students’ performance has been a topic of study in the fields of economics (e.g., Holmlund & Sund, 2008), education (e.g., Aslam & Kingdon, 2011), and public administration (e.g., Keiser et al., 2002) for decades. Theories from these diverse fields of research suggest that the match between teachers’ gender and students’ gender can result in positive effects on students’ performance. Positive effects of student–teacher gender congruence are suggested to be the result of three, possibly co-occurring, explanations (i.e., mechanisms): teachers’ preferences, teachers’ gender stereotypes, and students’ role model perception (Aslam & Kingdon, 2011). The preference explanation implies that teachers reward students who have the same gender as them with higher grades (Aslam & Kingdon, 2011). In addition to grading, teachers are assumed to pay more attention and to give more positive feedback to students of the same gender (e.g., Grissom et al., 2015). In public administration, these intentional and active behaviors of teachers towards students they represent (students of the same gender) are referred to as active representation (Riccucci & Van Ryzin, 2017). The gender stereotype explanation is similar to the preference explanation but suggests that the favoring of students of the same gender happens implicitly and unconsciously based on teachers’ gender stereotypes (Doornkamp et al., 2022). Gender stereotypes are socially constructed ideas that describe what men and women are like and should be like (Ellemers, 2018). Based on gender stereotypes, male students are described as being naturally talented in math while female students are described as being naturally talented in languages. As a result, teachers may unconsciously have different expectations of and behaviors towards male and female students in different subjects. The gender stereotypes of male and female teachers can vary. Teachers in counter-stereotypical subjects (e.g., female teachers in STEM and male teachers in languages) tend to have weaker gender stereotypes than their counterparts (male teachers in STEM and female teachers in languages) (Doornkamp et al., 2022; Martin & Dinella, 2012; Smeding, 2012). As a result, female teachers in STEM and male teachers in languages may have more equal expectations of male and female students in their subjects (Denessen et al., 2022; Muntoni & Retelsdorf, 2018; van den Bergh et al., 2010). As a consequence, these teachers may show less biased behaviors which may benefit the students they represent. Lastly, the role-model explanation takes the perspective of the student and argues that students can perceive teachers with the same gender as a role model which stimulates behaviors that positively affect students’ performance (Aslam & Kingdon, 2011). In public administration this mechanism is referred to as symbolic representation (Riccucci & Van Ryzin, 2017).

Although student–teacher gender congruence is expected to lead to positive effects, empirical evidence for this association is mixed (for cross-national comparisons see An et al., 2021; Cho, 2012). Positive relations between student–teacher gender congruence and students’ performance were found for both females and males (Dee, 2007; Muralidharan & Sheth, 2016), and only for females and not for males (Lee et al., 2019; Zhang, 2019). Negative relations between student–teacher gender congruence and students’ performance have also been found for females and not for males (Dee, 2007),^1^ and only for males and not for females (Doornkamp et al., 2023). Finally, some studies did not find a relation between student–teacher gender congruence and students’ performance at all (Holmlund & Sund, 2008; Neugebauer et al., 2011). The large variety in the countries, education systems, education levels, and education years (grade) in which studies on the topic of student–teacher gender congruence and student performance where conducted may explain the inconsistency in the study’s findings.

### The role of context

These ambiguous findings may imply that there are conditions under which positive as well as negative relations between student–teacher gender congruence and students’ performance emerge (An et al., 2021). To our knowledge, thus far the role of different educational contexts in effects of student–teacher gender congruence has not been explored. Although public administration scholars identified organizational (Dhillon & Meier, 2022; Song, 2018) and political conditions (An et al., 2021) for positive effects of student–teacher gender congruence to occur, still little is known about the educational contexts in which positive effects of student–teacher gender congruence emerge.

Effects of student–teacher gender congruence are assumed to emerge in educational contexts in which gender is perceived as salient, i.e., important, by students and teachers (e.g., Keiser et al., 2002). Gender becomes a salient characteristic when a gender-related concept of self and/or others is activated (Bem, 1981; Palan, 2001). Gender-related concepts can be activated in contexts in which there is an observable differentiation between men and women, for instance by dress codes in which women have to wear skirts and men have to wear ties, or when differential language or labels are used to refer to men and women (Hilliard & Liben, 2010). When men and women have different dress codes or are referred to differently, gender becomes an important characteristic for people’s identity (what is appropriate to wear for me as a man/woman) and social interactions (the way people speak to me as a man/woman). Previous research indicates that students’ gender stereotypes can become stronger in educational settings in which the gender dichotomy is emphasized (Bigler, 1995). As a result, gender becomes more salient and the association between student–teacher gender congruence and performance may be strengthened. Further, gender can become salient when a policy is directed to benefit women as a group (Keiser et al., 2002). For instance, a policy directed to improve female participation in STEM attracts attention to gender in relation to STEM. As a result, female students and teachers may be more aware of their gender in the STEM context. The introduction of the policy may motivate female teachers in STEM to encourage female students to improve their participation in STEM. At the same time, the policy may improve female students’ awareness on the underrepresentation of women in STEM which may increase their admiration and perception of their female teacher. As a result of teachers’ efforts and students’ changed attitudes, female students performance may improve. Similarly, gender may become salient when an issue is identified as a gender issue through the political process (Keiser et al., 2002). For instance, the feminization of the education system recently entered the political agenda (Holmlund & Sund, 2008; Neugebauer et al., 2011). The political attention to and problematization of lack of gender representation for male students in education may improve students’ appreciation of teachers of the same gender. At the same time, male teachers may be motivated to improve their efforts for male students contributing to male students’ performance.

The differentiation between males and females often happens on the basis of gender stereotypes. Gender stereotypes are socially constructed ideas about what men and women are like, and what men and women should be like regarding their appearances, behaviors, and capabilities (Ellemers, 2018). Gender stereotypes can differ across social contexts (e.g., Miller et al., 2015). In schools, the extent to which gender salience is perceived by teachers and/or students may vary for males and females across subjects with gender stereotypes connected to it. Males and females’ capabilities in STEM and languages are gender stereotyped as different: males are gender stereotyped as good at STEM and bad at languages (Schmenk, 2004), while females are gender stereotyped as good at languages and bad at STEM (Brown & Stone, 2016). Negative gender stereotypes (i.e., bad at) are generally easier to internalize and more resistant to change than positive gender stereotypes (Baumeister et al., 2001). Therefore, gender can become a salient characteristic for the group that is stereotypically known for its performance deficits (Beilock et al., 2010; Spencer et al., 2016; Starr & Simpkins, 2021). As a result, gender may be relatively salient for females in STEM and for males in languages. Accordingly, effects of student–teacher gender congruence are likely to benefit female students’ performance in STEM and male students’ performance in languages.

Student–teacher gender congruence positively affects students’ performance through the behaviors of teachers and/or students. Female teachers in STEM subjects and male teachers in language subjects may have experienced social barriers as a result of gender stereotypes throughout the process of becoming a female teacher in STEM or a male teacher in languages. Because of these personal experiences, female STEM teachers and male language teachers may reward students of the same gender with more points for their answers or give students of the same gender more positive reinforcements (i.e., compliments) in order to reduce social barriers and encourage students of the same gender to participate in the subjects. Further, female role models in STEM fields (Herrmann et al., 2016), and perhaps also male role models in language fields (DUO, 2022), are limited. As a result, female STEM teachers and male language teachers may function as important role models for female students in STEM and male students in languages, which can positively affect students’ attitudes, behaviors, and ultimately, their performance (Hermann et al., 2016; Solanki & Xu, 2018). Based on the above we hypothesize:

#### H1a

Student–teacher gender congruence is positively related to female students’ performance in math and physics.

#### H1b

Student–teacher gender congruence is positively related to male students’ Dutch language and French language performance.

Apart from the type of subject, the extent to which gender stereotypes connected to the subjects play a role in effects of student–teacher gender congruence may differ across educational contexts including students’ educational levels, schools’ religiousness, and schools’ location. Previous studies on socioeconomic status (SES) showed that stereotypical gender roles and behaviors are more pronounced in segments of the population with lower SES compared to segments with higher SES (Trusty et al., 2000). In education, students from lower SES segments were found to perceive higher career barriers, including social barriers that stem from gender stereotypes, than students from higher SES segments (Turner et al., 2019). As SES and educational level are closely related concepts (educational attainment is an important indicator for SES and is often used as one of the measures for SES (e.g., Saifi & Mehmood, 2011), the same reasoning might apply to students’ educational level. Students in lower educational levels might perceive gender as more salient. Students’ educational pathways in the Netherlands seem to support this idea. The educational pathways of Dutch students reveal gender stereotypic choices: male students tend to choose education in STEM and female students tend to choose education in (health)care (Onderwijsraad, 2019). These gendered choices are the largest among the lowest educational levels (Bügel et al., 2011), possibly implying that gender stereotypes play a more important role in lower educational levels. In this context in which gender stereotypes are assumed to be relatively strong, female teachers in STEM and male language teachers may feel a stronger need to reduce social barriers that stem from gender stereotypes and encourage students of the same gender to participate in the subject, reinforcing effects of student–teacher gender congruence. Further, access to contra-gender stereotypical role models may be even more limited among lower educational levels. The few contra-stereotypical role models in this context may be of greater importance, increasing student–teacher gender congruence effects. We therefore hypothesize:

#### H2

The associations between student–teacher gender congruence and students’ performance are stronger at lower educational levels.

Similarly, gender stereotypes in religious schools might be stronger than gender stereotypes in non-religious schools. Religiousness is often associated with more traditional views on roles of men and women (de Vries et al., 2022). Traditional views on males and females may result in relatively strong gender stereotypes. Indeed, previous research showed that students in religious schools hold stronger gender stereotypical beliefs than students from non-religious schools (Schulze & Tomal, 2005). As a result, we expect effects of student–teacher gender congruence to be stronger in religious schools.

#### H3

The associations between student–teacher gender congruence and students’ performance are stronger in religious schools.

**Fig. 1:**
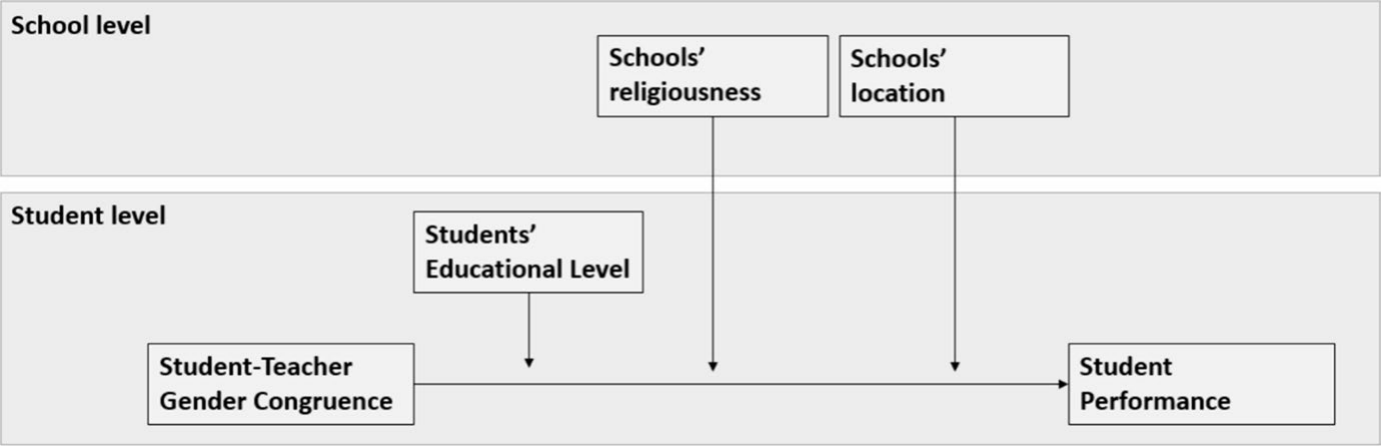
Research model

Finally, effects of student–teacher gender congruence might be stronger in schools in less-populated areas compared to heavily populated areas. In general, residents in rural areas express more conservative attitudes and follow more traditional values compared to residents in urban areas (Istenič, 2007). As people in heavy populated areas generally tend to be more progressive and heterogenous, people living in heavy populated areas have a bigger chance of meeting contra-gender stereotypical role models than people living in less-populated areas. Similarly, because of larger population sizes in heavy populated areas, people living in these areas have a bigger chance of meeting contra-gender stereotypical role models. As a consequence, individuals in less-populated areas may hold stronger gender stereotypical beliefs than individuals in heavily populated areas. Indeed, population size and gender stereotypical beliefs were found to be negatively related (Garcia-Retamero et al., 2011). Therefore, effects of student–teacher gender congruence might be stronger in less-populated areas (Dhillon & Meier, 2022). The research model is visualized in Fig. 1.

#### H4

The associations between student–teacher gender congruence and students’ performance are stronger in schools located in less populated areas.

## Method

### Procedure

This study is part of the research project Girls in Science running from 2017 until 2022 in the Netherlands which examines gender socialization in the family and school context. The data for this study were collected between December 2019 and February 2021 through secondary schools’ student administration systems. In collaboration with The Implementation Group (TIG) we were able to extract relevant data from these systems. TIG is an organization that develops administrative tools for secondary schools to inform schools about patterns in their student data. Approximately half of all secondary schools in the Netherlands are affiliated with TIG. Through this affiliation TIG has access to a great amount of secondary school data.

The affiliated schools were asked to participate in the research. Participation involved giving consent to TIG to transfer a selection of anonymized administrative data to the researchers for research purposes. The data included students’ gender, educational level per year, subject choice per year, student performances (grades) per year in four subjects (math, physics, Dutch language, and French language), and corresponding teachers’ gender for the subjects per year over the period 2011 until 2021. Additionally, school characteristics including school denomination, school structure, and level of urbanity were collected based on open access data. Participating schools received the results of the study and an invitation to a symposium on the role of gender in education. Seventeen percent of schools affiliated with TIG participated in the research resulting in an unique dataset with data on student performance throughout school careers of 403.984 students nested in 195 secondary schools starting from 2011 until 2021. For this research, we used a subset of this dataset.

### Sample

This study focused on the last year of lower secondary education when all students still participate in all subjects. In this year, students choose the subjects they want to participate in higher secondary education and ultimately want to graduate in. The year in which Dutch students choose their subjects and transfer from lower to higher secondary education is dependent on the educational level they are in. Students in the educational level that prepares for vocational education (vocational track) have four years of secondary education and choose their subjects in their second year. Students in the educational level that prepares for applied universities (higher vocational track) as well as students in the educational level that prepares for university (scientific track) have respectively five and six years of secondary education and choose their subjects in their third year. As a result, this study included data from students in year 2 (vocational track) and year 3 (higher vocational track and scientific track) and excluded data from all other years.

Some Dutch secondary schools provide combinations of educational levels in lower secondary education. In this study we excluded students who combined or switched educational level. We included students’ that started secondary education in 2011 until 2021. We included only those students with scores on all relevant variables per subject, resulting in *N* = 83,271 for math (51% male), *N* = 65,902 for physics (49% male), *N* = 87,293 for Dutch language (49% male), and *N* = 58,242 for French language (48% male).

### Measures

#### Student–teacher gender congruence

The independent variable refers to student and teacher having the same gender (0 = no gender congruence, 1 = gender congruence). Students’ and teachers’ gender were administrated in the secondary school administration system as male, female or neutral. As there were few students’ (*n* = 2) and teachers’ (*n* = 133) that were administrated as neutral, analyses on the effects of student–teacher gender congruence for the non-binary group were not possible. Students’ and teachers’ administrated as neutral were therefore regarded as missing data.

The distribution of male and female teachers across the subjects is skewed but similar for male and female students (see Table 1). The percentages of students with a female teacher for the subjects are: math 37%, physics 26%, Dutch language 69%, and French language 79%. The distribution of male and female teachers over the subject in our sample is comparable with the distribution in the Netherlands (math 45% female, physics 21% female, Dutch language 74% female, and French language 82% female (DUO, 2022)). The skewed distribution is not expected to introduce bias, because teachers do not select their students (or vice versa).

**Table 1:** Range, means, standard deviations, and distributions of central variables

	Range	Mean (SD) and distribution		
		Full sample	Male students	Female students
*Math*		*N* = 83,271	*n* = 41,223	*n* = 42,048
1 Grade	1–10	6.51 (1.11)	6.42 (1.08)	6.59 (1.13)
2 Gender Congruence	0, 1	50/50	37/63	63/37
3 Educational Level	1, 2, 3	46/27/27	48/27/25	44/27/29
4 Schools’ Religiousness	0, 1, 2	26/61/13	25/61/13	26/60/13
5 Schools’ Location	0, 1	39/61	39/61	39/61

*Physics*		*N* = 65,902	*n* = 32,289	*n* = 33,613
1 Grade	1–10	6.50 (0.96)	6.52 (0.94)	6.48 (0.97)
2 Gender Congruence	0, 1	50/50	25/75	74/26
3 Educational Level	1, 2, 3	34/33/33	37/33/31	32/33/35
4 Schools’ Religiousness	0, 1, 2	25/60/15	25/60/15	25/59/16
5 Schools’ Location	0, 1	34/66	35/65	34/66

*Dutch*		*N* = 87,293	*n* = 43,157	*n* = 44,136
1 Grade	1–9.8	6.55 (0.74)	6.33 (0.70)	6.77 (0.70)
2 Gender Congruence	0, 1	50/50	69/31	31/69
3 Educational Level	1, 2, 3	45/27/28	47/27/26	42/27/30
4 Schools’ Religiousness	0, 1, 2	26/61/13	26/61/13	26/61/13
5 Schools’ Location	0, 1	38/62	38/62	38/62

*French*		*N* = 58,242	*n* = 27,808	*n* = 30,434
1 Grade	1–10	6.53 (1.11)	6.21 (1.08)	6.83 (1.06)
2 Gender Congruence	0, 1	49/51	79/21	21/79
3 Educational Level	1, 2, 3	24/37/39	25/38/38	23/36/41
4 Schools’ Religiousness	0, 1, 2	26/59/15	27/58/15	26/60/14
5 Schools’ Location	0, 1	32/68	32/68	32/68

Distributions are presented in percentages. Gender congruence 0 = no congruence, 1 = congruence; Educational level 1 = vocational track, 2 = higher vocational track, 3 = scientific track; Schools’ religiousness 0 = not religious, 1 = religious roots, 2 = practicing religious; Schools’ location 0 = suburban, 1 = urban

#### Student performance

The dependent variable refers to students’ grades for math, physics, Dutch language, and French language. Students’ grades were obtained from the calculated averages ranging from 1.0 to 10.0 administrated in the secondary school administration systems. The calculated averages are students’ average final grade per year. The calculated end averages in 2021 represent the current average grades of the students in that year (as the data was subtracted from the student administration systems in 2021). Grades between 0.0 and 1.0 were regarded as missing data (*n* = 9).

#### Students’ educational level

The first moderating variable, educational level, was obtained from students’ ‘ILT-code’ (Integral Student Count) that was administrated in the secondary school administration systems. The ILT-code is a governmental code that includes students’ educational level. The ILT-code variable was recoded into the educational variable with 1 = vocational track, 2 = higher vocational track, and 3 = scientific track.

#### Schools’ religiousness

The second moderating variable, schools’ religiousness, was deduced from schools’ websites. Schools with an open character not referring to any religion or explicitly stating not to be a religious school were coded as 0 = not religious. Schools with religious roots referring to religious values but explicitly stating to be open to students from any (non) religious background were coded as 1 = religious roots. Schools that explicitly stated that they practice religion in their education were coded as 2 = practicing religious. Students in schools whose degree of religiousness could not be coded based on the information provided on their website were regarded as missing data (*n* = 14,719).

#### Schools’ location

The third moderating variable refers to the address density per squared kilometer of the schools’ location. We obtained data on address density in the postal code of the participating schools using open governmental data. We recoded level of urbanity variable in 0 = suburban, less-populated area (less than 1500 addresses), and 1 = urban, heavy populated area (more than 1500 addresses), based on the breaking point between ‘moderate’ and ‘strong urban’ governmental classification (CBS, 2022).

### Data analyses

The data inspection and further analyses were conducted using R statistical software. To check for normal distributions Q–Q plots were inspected. The normality checks indicated that some variables had heavy tails, however this type of minor divergence from normality is unlikely to cause any major problems given the large sample sizes (Faraway, 2015; Fox, 2015; Lumley et al., 2002). After checking the assumptions, which were not violated, no clear signs of outliers were observed. This in combination with the large sample sizes and the scales of the variables, outliers are assumed not to be influential (Fox, 2015).

To test our hypotheses, we performed a series of multilevel ANOVAs of increasing complexity using the lme4 package (Bates et al., 2015). We performed four separate multilevel ANOVAs for student performance in math, physics, Dutch language, and French language. We used multilevel ANOVAs to correct for school differences. Further, we performed the multilevel ANOVAs to be able to observe male and female students’ mean grades (student performance) for the teacher gender-congruent versus incongruent condition. To test hypothesis 1, an F-test is performed to assess if students’ mean grades of the student–teacher gender dyad were equal. Additional post hoc tests were conducted to examine whether the mean grades of students with teachers of the same gender were significantly different from students without teachers of the same gender (i.e., teachers of the other gender). To test the robustness of the results for hypothesis one, we conducted the analyses on the largest possible sample size per subject (results reported in Appendix A). For hypotheses H2–H4, the data set was split based on students’ gender to ease the interpretation of the student–teacher gender congruence variable and its interaction with the context variables. Therefore, we performed eight separate multilevel ANOVAs to be able to observe male and female students’ mean grades for the teacher congruent and teacher incongruent condition for different educational contexts. First, F-tests were performed to see whether interaction terms between student–teacher gender congruence and the contextual factors educational level (hypothesis 2), schools’ religiousness (hypothesis 3), and schools’ location (hypothesis 4) held significant information. Thereafter, post hoc tests were performed to test whether student performance differed significantly between the gender congruent and gender incongruent condition for each contextual factor.

## Results

### Student–teacher gender congruence and students’ performance

Table 1 presents the range, means, standard deviations, and distributions for student–teacher gender congruence, students’ performance, and the contextual variables in math, physics, Dutch language, and French language. Table 2 presents the means for the different student–teacher gender combinations. Both Tables 1 and 2 show that female students had higher average grades than male students in math, Dutch language, and French language, but not in physics.

**Table 2:** Student–teacher gender congruence and student performance

	Male student		Female student	
	Male teacher	Female teacher	Male teacher	Female teacher
Math *N* = 83,271	6.36^a^	6.36^a^	6.51^b^	6.56^c^
Physics *N* = 65,902	6.46^a^	6.45^a^	6.39^b^	6.47^a^
Dutch *N* = 87,293	6.35^a^	6.28^b^	6.75^c^	6.73^c^
French *N* = 58,242	6.22^a^	6.12^b^	6.78^c^	6.77^c^

Means with different superscripts in rows are significantly different from each other on *p* < .001, Standard Errors range from 0.03 to 0.04

Further, Table 2 shows that in math female students with female teachers had significantly higher grades than female students with male teachers. Male students’ math performance did not differ for the gender of their teachers. In physics, male students with male teachers, male students with female teachers, and female students with female teachers had (approximately) the same average grades, only female students with male teachers had significantly lower grades. In Dutch language and French language, female students’ performance did not differ for the gender of their teacher. However, male students with male teachers had significantly higher grades than male students with female teachers in both Dutch language and French language. Based on these results, hypothesis 1 should be accepted: student–teacher gender congruence was positively related to performance in math and physics for female students (H1a) and performance in Dutch language and French language for male students (H1b). Robustness checks on the largest possible samples confirmed the results (see Appendix A).

### The role of context

In the next step we tested whether students’ educational level, schools’ religiousness, and schools’ location moderated the relation between student–teacher gender congruence and students’ performance. Tables 3 and 4 present the results of the moderation analyses for male (Table 3) and female (Table 4) students’ performance.

**Table 3:** Multilevel ANOVA of male student–teacher gender congruence on students' performance, moderated by students’ educational level, schools’ religiousness, and schools’ location with Post Hoc test

	Math		Physics		Dutch		French	
	Model 1	Model 2	Model 1	Model 2	Model 1	Model 2	Model 1	Model 2
Gender Congruence	0.34	1.65	0.35	0.06	85.77***	71.77***	20.13***	2.17
Educational Level	285.67***	227.08***	638.34***	567.64***	462.64***	439.81***	426.79***	303.81***
Religiousness	1.29	1.34	2.20	2.77	2.05	2.17	0.23	0.50
Location	0.20	0.19	0.31	0.41	2.28	0.88	0.40	1.09
GCxEduLevel		1.94		49.50***		2.12		1.59
GCxReligiousness		6.26**		8.14***		1.72		3.45*
GCxLocation		0.00		1.49		12.88***		8.70**
Conditional R2	0.08	0.08	0.14	0.15	0.16	0.16	0.14	0.14

	Mean	Difference	Mean	Difference	Mean	Difference	Mean	Difference
GC0x EduLevel1	6.32		6.48		6.26		5.90	
GC1x EduLevel1	6.35	− 0.03	6.37	0.11***	6.34	− 0.08***	5.98	− 0.08*
GC0xEduLevel2	6.26		6.16		6.17		6.04	
GC1xEduLevel2	6.24	0.01	6.34	− 0.18***	6.23	− 0.06***	6.05	− 0.01
GC0x EduLevel3	6.56		6.84		6.44		6.43	
GC1x EduLevel3	6.61	− 0.04	6.76	0.08**	6.54	− 0.10***	6.44	− 0.01
GC0xReligiousness0	6.29		6.42		6.21		6.11	
GC1xReligiousness0	6.36	− 0.07**	6.40	0.02	6.27	− 0.06***	6.19	− 0.08*
GC0xReligiousness1	6.40		6.40		6.31		6.15	
GC1xReligiousness1	6.37	0.03	6.48	− 0.08***	6.39	− 0.08***	6.23	− 0.08***
GC0xReligiousness2	6.46		6.66		6.34		6.10	
GC1xReligiousness2	6.47	− 0.01	6.58	0.08*	6.46	− 0.11***	6.05	0.05
GC0xLocation0	6.37		6.46		6.25		6.10	
GC1xLocation0	6.39	− 0.02	6.48	− 0.01	6.36	− 0.11***	6.08	0.03
GC0xLocation1	6.39		6.53		6.33		6.14	
GC1xLocation1	6.41	− 0.02	6.50	0.02	6.38	− 0.05***	6.23	− 0.09***

In the top half, F-Values of the Multilevel ANOVA are reported, in the bottom half the gender congruent condition was post hoc tested against the gender incongruent condition (i.e., second row vs first row), ***p < .001, **p < .01, *p < .05

**Table 4:** Multilevel ANOVA of female student–teacher gender congruence on students' performance, moderated by students’ educational level, schools’ religiousness, and schools’ location with Post Hoc test

	Math		Physics		Dutch		French	
	Model 1	Model 2	Model 1	Model 2	Model 1	Model 2	Model 1	Model 2
Gender Congruence	6.95**	2.54	14.12***	4.78*	17.89***	8.09**	1.05	0.26
Educational Level	427.95***	377.66***	948.43***	760.20***	711.17***	626.73***	548.62***	380.29***
Religiousness	1.18	1.07	3.95*	3.93*	2.39	2.43	0.97	0.92
Location	0.96	1.40	0.16	0.08	0.01	0.01	1.58	0.86
GCxEduLevel		0.88		36.96***		2.09		1.98
GCxReligiousness		1.35		0.76		1.12		0.28
GCxLocation		12.38***		1.45		0.00		4.76*
Conditional R2	0.09	0.09	0.16	0.16	0.19	0.19	0.13	0.13

	Mean	Difference	Mean	Difference	Mean	Difference	Mean	Difference
GC0x EduLevel1	6.51		6.38		6.72		6.63	
GC1x EduLevel1	6.55	− 0.04*	6.54	− 0.16***	6.72	0.01	6.57	0.06
GC0xEduLevel2	6.34		6.23		6.62		6.63	
GC1xEduLevel2	6.37	− 0.03	6.12	0.11***	6.58	0.04**	6.63	− 0.00
GC0x EduLevel3	6.79		6.75		6.95		7.05	
GC1x EduLevel3	6.79	− 0.00	6.81	− 0.07**	6.91	0.03*	7.07	− 0.02
GC0xReligiousness0	6.48		6.33		6.66		6.75	
GC1xReligiousness0	6.51	− 0.03	6.39	− 0.06	6.64	0.02	6.76	− 0.01
GC0xReligiousness1	6.52		6.42		6.80		6.84	
GC1xReligiousness1	6.58	− 0.05***	6.43	− 0.01	6.76	0.04***	6.83	0.01
GC0xReligiousness2	6.63		6.60		6.83		6.71	
GC1xReligiousness2	6.63	0.01	6.65	− 0.04	6.81	0.02	6.68	0.03
GC0xLocation0	6.56		6.47		6.77		6.79	
GC1xLocation0	6.63	− 0.07**	6.49	− 0.02	6.74	0.03	6.82	− 0.03
GC0xLocation1	6.53		6.43		6.76		6.75	
GC1xLocation1	6.51	0.02	6.49	− 0.06**	6.73	0.03**	6.70	0.05*

*Note:* In the top half, F-Values of the Multilevel ANOVA are reported, in the bottom half the gender congruent condition was post hoc tested against the gender incongruent condition (i.e., second row vs first row), ***p < .001, **p < .01, *p < .05

As expected, student–teacher gender congruence explained variance in student performance for females in math and physics and for males in Dutch language and French language. Additionally, Table 4 shows that student–teacher gender congruence explained variance in female students’ Dutch language performance. Based on our hypotheses we expected student–teacher gender congruence to matter in particular in the lowest educational level (vocational track), in religious schools, and in suburban located schools. We therefore expected (the largest) negative significant difference between the incongruent and congruent condition in the bottom half of Tables 3 and 4 for the vocational track, religious schools, and suburban located schools. We will discuss the results for each contextual factor separately.

#### Students’ educational level

**Fig. 2:**
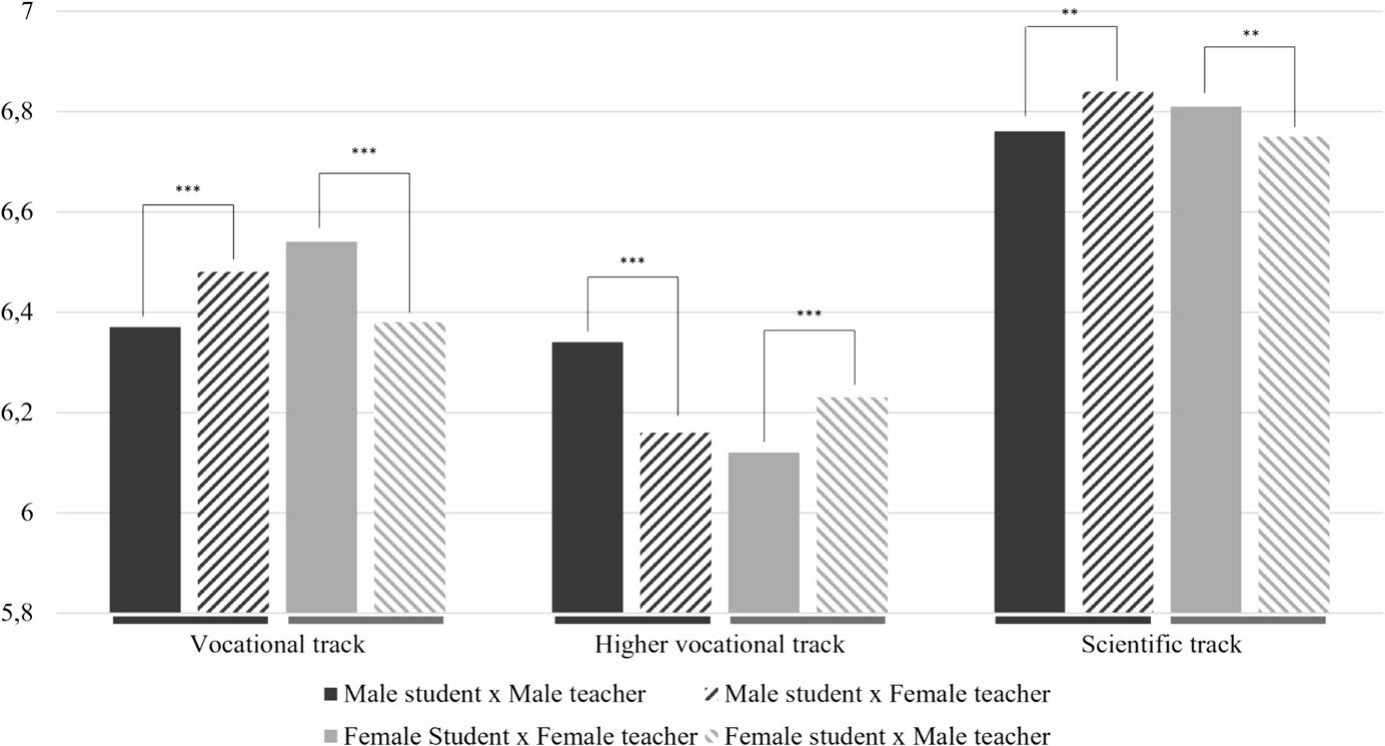
Student–teacher gender congruence and educational level on students’ physics performance

The interaction between student–teacher gender congruence and educational level was not significant in math, Dutch language, and French language indicating that the relation between student–teacher gender congruence and students’ performance did not differ for students’ educational level in these subjects. However, in physics, the interaction between gender congruence and educational level explained variance in both males’ and females’ physics performance. For males, the post hoc analyses revealed that gender congruence was related to higher grades in the higher vocational track while gender congruence was related to lower grades in the vocational track and the scientific track. For females, gender congruence was related to higher grades in the vocational and scientific track, whereas gender congruence was related to lower grades in the higher vocational track. These findings are visualized in Fig. 2. In all, hypothesis 2 can only be accepted in the subject physics for female students in the vocational and scientific track and for male students in the higher vocational track, in all other contexts the hypothesis should be rejected.

#### Schools’ religiousness

**Fig. 3:**
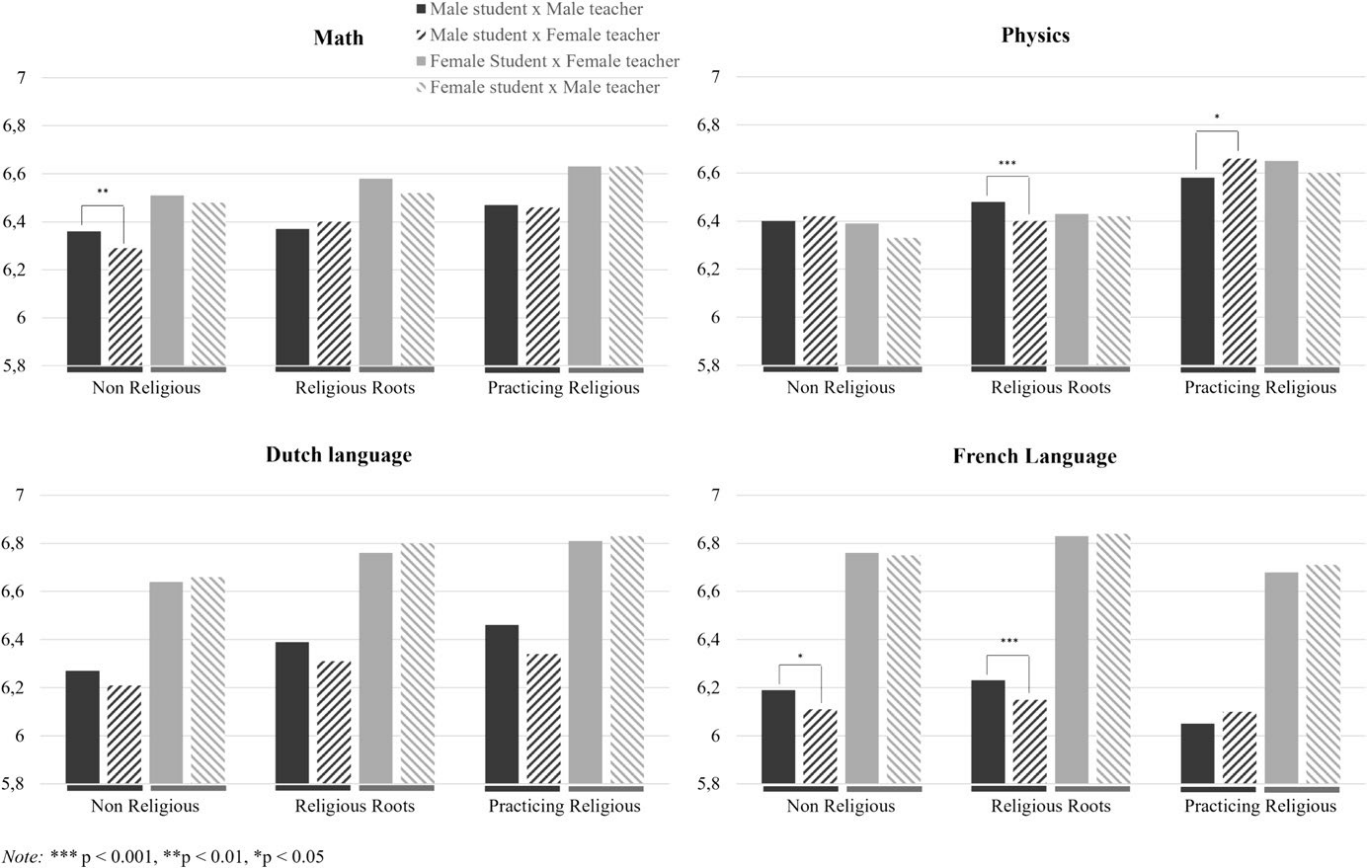
Student–teacher gender congruence and schools’ religiousness on students’ performance by subject

The interaction between student–teacher gender congruence and schools’ religiousness was significant for males in math, physics, and French language. The interaction between gender congruence and religiousness was not significant for male students in Dutch language, nor for female students in any of the subjects. For males in math, the post hoc analyses revealed that in non-religious schools, male students with male teachers had slightly higher average grades than male students with a female teacher. In schools with religious roots and in schools that practice religion, gender congruence was not associated with male students’ math performance. In physics, gender congruence was associated with higher grades for males in schools with religious roots whereas gender congruence was associated with lower grades in schools that practice religion. In non-religious schools gender congruence did not affect male students’ physics grades. In French language, gender congruence was related to higher grades for males in non-religious schools and in schools with religious roots. Gender congruence was not related to male students’ performance in religious schools. The findings are visualized in Fig. 3. In all, hypothesis 3 should be rejected as student–teacher gender congruence was not associated with higher average grades in any of the subjects for neither male students nor female students.

#### Schools’ location

**Fig. 4:**
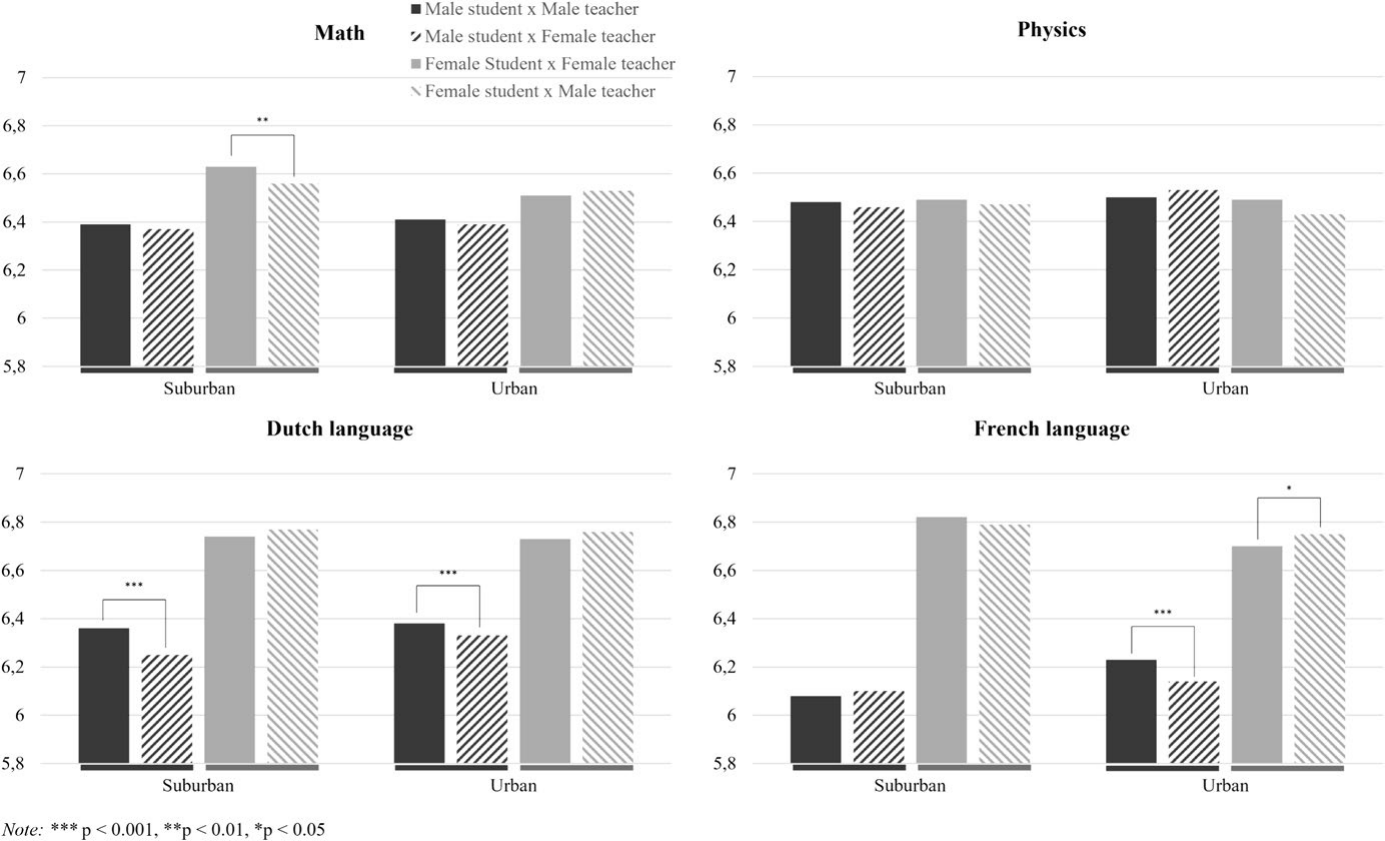
Student–teacher gender congruence and schools’ location on students’ performance by subject

The interaction between student–teacher gender congruence and schools’ location was significant for male students in Dutch language and French language and for females in math and French language. For males in Dutch language, the post hoc analyses showed that gender congruence was related to higher grades in suburban-located schools. Gender congruence was not related to male students’ Dutch language performance in schools that are located in urban areas. In French language, gender congruence was related to higher grades in schools located in urban areas, and not in schools located in suburban areas. For females in math, student–teacher gender congruence was related to higher grades in schools located in suburban areas and not in schools located in urban areas. In French language, gender congruence in schools in urban areas was related to lower grades. Gender congruence was not related to female students’ performance in schools in suburban areas. The findings are visualized in Fig. 4. In all, hypothesis 4 should only be accepted for male students in Dutch language and female students in math.

## Discussion and conclusion

This study tested the relation between student–teacher gender congruence and students’ performance and examined the role of students’ educational level, schools’ religiousness, and schools’ location. We found that student–teacher gender congruence was positively related to female students’ performance in math and physics and to male students’ performance in Dutch language and French language. Further, we found differential roles for different educational contexts for males and females and across subjects.

### Gender congruence and performance

Our results support previous research in the fields of economics, education, and public administration that found positive effects of student–teacher gender congruence for both males and females (e.g., Dee, 2007), but adds to this literature that positive effects of student–teacher gender congruence emerge in different subjects for male and female students. We found that female students benefited from female teachers in math and physics but not in Dutch language and French language, and that male students benefited from male teachers in Dutch language and French language but not in math and physics. We argued that this differential role of student–teacher gender congruence for males and females could be the result of differences in the extent to which males and females perceived gender salience in given subjects (Doornkamp et al., 2021; Keiser et al., 2002). We suggested that gender was salient for female teachers and students in STEM and for male teachers and students in languages because these are the contexts in which these groups are stereotypically thought to have performance deficits (e.g., Spencer et al., 2016). In these contexts, effects of student–teacher gender congruence may have emerged through teachers’ and/or students’ attitudes and behaviors (Aslam & Kingdon, 2011; Zhang, 2019). For instance, from the teacher perspective, female teachers in STEM and male teachers in languages might have tried to (intentionally or unintentionally) reduce social barriers that stem from gender stereotypes by rewarding students of the same gender with higher grades or by giving more positive feedback to them (Aslam & Kingdon, 2011; Grissom et al., 2015). From a student perspective, female teachers in STEM and male teachers in languages might have functioned as important contra-gender stereotypical role models affecting students’ attitudes, behaviors, and ultimately performance (e.g., Solanki & Xu, 2018).

An additional remark with regard to these findings should be made. In physics, female student–teacher gender congruence and performance was not *positively* related as female students with a female teacher did not perform better than average. Instead, female students with a female teacher performed as well as male students with both male and female teachers. Female students with a male teacher performed worse than the average (worse than all other groups) indicating that student–teacher gender *incongruence* had a negative effect on female students’ physics performance. Negative effects of student–teacher gender incongruence have been found in previous research (Dee, 2007; Solanki & Xu, 2018). However, it should be noted that in contrast to our finding, these studies found that the negative effects of gender incongruence occurred simultaneously with positive effects of gender congruence (Dee, 2007; Solanki & Xu, 2018). Negative effects of gender incongruence could have stemmed from male teachers’ differential treatment of male and female students or from negative attitudes or self-beliefs in physics in female students as a result of having a male physics teacher. Perhaps, male physics teachers had strong associations between physics and masculinity (Doornkamp et al., 2022). Therefore, male physics teachers might have underestimated female students’ capabilities in physics which could have led to stricter grading or generating less positive reinforcements for female students (Denessen et al., 2022). From a student perspective, female students’ performance might have been weakened by having a male physics teacher through the mechanism of stereotype threat (Kapitanoff & Pandey, 2017). Female students’ physics performance could have been suppressed by the stereotypical belief that women are bad at physics (Marchand & Taasoobshirazi, 2013). Whereas female physics teachers could have reduced effects of stereotype threat on female students’ performance by being a role model, male physics teachers could not (Kapitanoff & Pandey, 2017).

### The role of context

We expected gender stereotypes to be relatively strong and therefore effects of student–teacher gender congruence to occur in lower educational levels (Trusty et al., 2000; Turner et al., 2019), in religious schools (Schulze & Tomal, 2005), and in schools located in less-populated areas (Garcia-Retamero et al., 2011). However, our results did not support the hypotheses. We found differential patterns for educational context for male and female students and across the subjects.

Evidence for the role of students’ educational level was only found in the subject physics and the role was opposite for male and female students. In the vocational and scientific track, gender congruence led to positive effects for female students’ performance while gender congruence led to negative effects for male students’ performance. In the higher vocational track our findings were opposite: gender congruence was related to better performance for male students and worse performance for female students. The contradictory findings across educational levels raise questions about the dissimilarities in students and teachers in different educational levels. For instance, the associations between and characteristics of students and teachers in the higher vocational track might be different from the associations between and characteristics of students and teachers in the other educational tracks. To gain more insight in possible differences between students and teachers in different educational tracks and its relation to effects of gender congruence, future research could use qualitative research methods to systematically compare students and teachers in different educational levels. Ethnographic research methods such as participation observation may provide these insights.

We found no role of schools’ religiousness for female students and the role of schools’ religiousness for male students was contrary to what we expected. Gender congruence was associated with better performance of male students in non-religious schools for the subjects math and French language, and in schools with religious roots for the subject physics. Only for male students in physics we found an effect in religious schools, but this effect was negative: gender congruence was associated with lower grades for male students. Perhaps, specific student characteristics that are related to school religiosity could have acted as a buffer against the role of gender stereotypes and student–teacher gender congruence effects. For instance, students in religious schools generally perform better than students in non-religious schools (Jeynes, 2002). Relatively good performance is associated with having more self-perceived ability (Marsh et al., 2005), and students with more self-perceived ability are found to be less susceptible for effects of student–teacher gender congruence (Eble & Hu, 2017).

For males in Dutch language and females in math, gender congruence led to better performance in less-populated areas and not in heavily populated areas which supported our hypothesis. These findings are similar to those of Dhillon and Meier (2022) who found positive gender congruence effects for females in math in rural areas. It should be noted that this finding was more complex as math performance of female students decreased when the female teacher percentage increased (Dhillon & Meier, 2022). In addition, in French language, gender congruence was associated with higher grades for males and lower grades for females in heavily populated areas. Further, schools’ location did not affect the relation between student–teacher gender congruence and performance in physics. These findings, and the other findings regarding the role of context, emphasize that effects of student–teacher gender congruence can differ in magnitude and direction in different contexts, but a clear pattern in these differential findings was not observed in this study.

### Limitations

Our findings should be interpreted within this study’s limitations. First, as a result of our large dataset very small differences in means can become statistically significant (Khalilzadeh & Tasci, 2017). Statistically significant effects smaller than 0.05 (on the 95% confidence interval) could be considered as practically insignificant. The results of our moderation analyses showed statistically significant mean differences smaller than 0.05 indicating that students with teachers of the same gender achieved less than 0.05 higher average grades than students with teachers of the other gender. This difference is very small and dissolves when average grades are round to 1 decimal, which is the case in most of Dutch secondary schools. Further, these effects might become statistically insignificant when student–teacher gender congruence effects are controlled for by predictors such as academic self-concept and student IQ which have been found to be important predictors of students’ performance (e.g., Wach et al., 2015). Nevertheless, the small differences in average grades for male and female students with a teacher of the same gender compared to a teacher of the other gender can have important consequences. In an education system in which calculated end averages are the basis for decisions with regard to students’ educational pathways (which is the case in the Netherlands), 0.1 can make the difference for passing a grade, moving up an educational level, or admission to further education.

Second, multicollinearity was introduced in some of our models and, as a consequence, variances were inflated. The models without interaction terms showed no signs of multicollinearity indicating that variance inflation was caused by the introduction of the interaction terms which does not create a multicollinearity problem (Disatnik & Sivan, 2016; Shieh, 2010).

Finally, our dataset relied on administrative data of secondary schools. The secondary schools’ administration systems were sometimes inconsistent in their registration. For instance, students’ grades can be registered under different columns with different terminology. Because of these inconsistencies in the administration of grades, several schools provided incomplete datasets. We asked participating schools to improve their registration and offered specific instructions and help for these improvements. Although some schools were able to improve their data for this study, not all schools were able to do so. Nevertheless, because we used registration data we still were able to collect a large amount of data without bias and/or systematic missings.

### Conclusion

In all, our study showed that student–teacher gender congruence not necessarily leads to better performance of male and female students. Our findings contribute to previous literature on the topic that having a teacher of the same gender leads to positive effects on student performance in secondary school subjects for the group of students that is confronted with a negative gender stereotype connected to that subject. Further, we build on previous literature by theorizing that these effects of student–teacher gender congruence could differ across educational contexts because of differences in the extent to which gender stereotypes play a role. Our results showed differential effects of student–teacher gender congruence in different contexts which did not correspond to our gender stereotype explanation. Thereby, our study encourages future research to further theorize and examine the role of context in effects of student–teacher gender congruence including factors such as socioeconomic status, schools’ size, and schools’ structure. Future research might want to use qualitative designs to explore the mechanisms that explain how context affects the role of gender in education.

Finally, our study contributes to education practice by providing empirical evidence for the role of students’ and teachers’ gender in student performance. The findings on the role of education context may invite school administrators and teachers to explore the extent to which gender plays a role in their school. The school administrators and teachers may examine their school administration data as well as trying to increase awareness about the strength of students’ and teachers’ gender stereotypes about men and women’s capabilities in STEM and languages in order to diminish or eliminate social barriers for students’ optimal performance.

## Appendix A: Robustness check Gender Congruence and Student Performance

See Table 5.

**Table 5:** Robustness check student–teacher gender congruence and student performance

	Male student		Female student	
	Male teacher	Female teacher	Male teacher	Female teacher
Math *N* = 97,082	6.35^a^	6.35^a^	6.50^b^	6.54^c^
Physics *N* = 76,286	6.44^a^	6.46^a^	6.38^b^	6.47^a^
Dutch *N* = 102,267	6.36^a^	6.28^b^	6.75^c^	6.74^c^
French *N* = 68,097	6.25^a^	6.15^b^	6.81^c^	6.79^c^

Means with different superscripts in rows are significantly different from each other on *p* < .001. Standard Errors range from 0.02 to 0.03

## Footnotes

1 Dee (2007) found positive and negative effects of student–teacher gender congruence on female students’ performance in different subjects.

## Declarations

### Confidentiality

All data collected remained confidential and were stored on a secured drive (only the researchers had access to this drive). All individual schools were assigned a unique identification number. The identification list that links schools’ unique identification numbers to school names and other identifying information has been deleted.

### Formal approval

Data collection and data management procedures were discussed with a data protection officer within the Faculty of Governance and Global Affairs, Leiden University. The Faculty of Governance and Global Affairs’ ethical clearance is in line with the General Data Protection Regulation (GDPR) and Leiden University’s code of conduct for scientific integrity. The formal ethical approval for this study was provided by the Ethics Committee of the Faculty of Governance and Global Affairs (Leiden University).
